# TRPC6-mediated ERK1/2 Activation Regulates Neuronal Excitability via Subcellular Kv4.3 Localization in the Rat Hippocampus

**DOI:** 10.3389/fncel.2017.00413

**Published:** 2017-12-20

**Authors:** Ji-Eun Kim, Jin-Young Park, Tae-Cheon Kang

**Affiliations:** Department of Anatomy and Neurobiology, Institute of Epilepsy Research, College of Medicine, Hallym University, Chuncheon, South Korea

**Keywords:** 4-AP, epilepsy, excitability ratio, hyperforin, paired-pulse response, seizure, U0126

## Abstract

Recently, we have reported that transient receptor potential channel-6 (TRPC6) plays an important role in the regulation of neuronal excitability and synchronization of spiking activity in the dentate granule cells (DGC). However, the underlying mechanisms of TRPC6 in these phenomena have been still unclear. In the present study, we investigated the role of TRPC6 in subcellular localization of Kv4.3 and its relevance to neuronal excitability in the rat hippocampus. TRPC6 knockdown increased excitability and inhibitory transmission in the DGC and the CA1 neurons in response to a paired-pulse stimulus. However, TRPC6 knockdown impaired γ-aminobutyric acid (GABA)ergic inhibition in the hippocampus during and after high-frequency stimulation (HFS). TRPC6 knockdown reduced the Kv4.3 clusters in membrane fractions and its dendritic localization on DGC and GABAergic interneurons. TRPC6 knockdown also decreased extracellular signal-regulated kinase 1/2 (ERK1/2) phosphorylation and the efficacy of 4-aminopyridine (4-AP) in neuronal excitability. An ERK1/2 inhibitor generated multiple population spikes in response to a paired-pulse stimulus, concomitant with reduced membrane Kv4.3 translocation. A TRPC6 activator (hyperforin) reversed the effects of TRPC knockdown, except paired-pulse inhibition. These findings provide valuable clues indicating that TRPC6-mediated ERK1/2 activation may regulate subcellular Kv4.3 localization in DGC and interneurons, which is cause-effect relationship between neuronal excitability and seizure susceptibility.

## Introduction

Transient receptor potential channel-6 (TRPC6) regulates Ca^2+^ influx and extracellular signal-regulated kinase 1/2 (ERK1/2) activity in the brain and thereby participates in synaptic plasticity and neuronal survival (Li et al., 2005; Du et al., 2010; Lin et al., 2013; Ko and Kang, 2017). In the hippocampus, TRPC6 is mainly expressed in dentate granule cells (DGC), CA3 pyramidal cells and γ-aminobutyric acid (GABA)-ergic interneurons (Kim et al., 2013; Nagy et al., 2013; Kim and Kang, 2015; Ko and Kang, 2017). Recently, we have reported that TRPC6 knockdown increases DGC excitability (field excitatory postsynaptic potential-population (fEPSP) spike component), synchronization of spiking activity in the population of DGC and the seizure susceptibility in response to pilocarpine. However, TRPC6 siRNA infusion also increases the paired-pulse inhibition in the dentate gyrus (Kim and Kang, 2015). Therefore, the functional role of TRPC6 in the regulation of the hippocampal excitability has been still unclear.

Dysfunction of voltage-gated K^+^ (Kv) channels is relevant to many neurological diseases, such as learning and cognitive impairment and epilepsy (Lawson, 2000; Wulff et al., 2009). Among the Kv channels, Kv4.3 underlies the somatodendritic A-type K^+^ currents (I_A_) that exhibit characteristic biophysical properties, such as rapid inactivation, fast recovery from inactivation and subthreshold activation (Jerng et al., 2004; Huang et al., 2005). Thus, Kv4.3 regulates the latency of first spike during depolarization, back-propagating action potentials and firing frequency (Hoffman et al., 1997; Malin and Nerbonne, 2001; Jerng et al., 2004; Kim et al., 2007; Truchet et al., 2012). Similar to TRPC6, Kv4.3 is present in interneurons, DGC and CA3 pyramidal cells (Rhodes et al., 2004). In interneurons, Kv4.3 also enables faster recovery from inactivation of A-type currents to promote stronger inhibitory control of firing during sustained repetitive stimuli (Bourdeau et al., 2011). Therefore, down-regulations of A-type K^+^ channels impair fast-spiking in interneurons, and subsequently increase excitability in principal neurons (Bernard et al., 2004; Birnbaum et al., 2004). Since the subcellular Kv4.3 localization is regulated by intracellular Ca^2+^ level and ERK1/2 activity (Kollo et al., 2006; Kim et al., 2007; Setién et al., 2013), it is likely that TRPC6 may regulate neuronal excitability via ERK1/2-mediated Kv4.3 translocation in DGC and interneurons. Therefore, in the present study, we explored this hypothesis, which has not been reported.

## Materials and Methods

### Experimental Animals and Chemicals

Male Sprague–Dawley (SD) rats (7 weeks old) were used. Animals were housed under standard conditions (23–25°C, 12 h light/dark cycle) with free access to food and water. All animal protocols were approved by the Administrative Panel on Laboratory Animal Care of Hallym University. All possible efforts were taken to avoid animals’ suffering and to minimize the number of animals used during the experiment. All reagents were obtained from Sigma–Aldrich (St. Louis, MO, USA), except as noted.

### TRPC6 siRNA and Drug Infusions

Under Isoflurane anesthesia (3% induction, 1.5%–2% for surgery and 1.5% maintenance in a 65:35 mixture of N_2_O:O_2_), animals were stereotaxically inserted a brain infusion kit 1 connected to an Alzet 1007D osmotic pump (Alzet, Cupertino, CA, USA) into the right lateral ventricle: 1 mm posterior to Bregma; 1.5 mm lateral to midline; 3.5 mm depth to the dural membrane. The infusion kit was sealed with dental cement. Using this infusion system, rats were given: (1) control siRNA; (2) TRPC6 siRNA; (3) vehicle; (4) U0126 (a selective ERK1/2 inhibitor, 25 μM); (5) TRPC6 siRNA + U0126; or (6) hyperforin (a TRPC6 activator, 6 μM). The sequence of TRPC6 rat siRNA (Genolution Pharmaceuticals, Inc., Seoul, South Korea) was 5′-GGAAUAUGCUUGACUUUGGAAUGUUUU-3′ (Kim and Kang, 2015; Ko and Kang, 2017). Non-targeting control siRNA sequence was 5′-GCAACUAACUUCGUUAGAAUCGUUAUU-3′. In our previous study (Ko and Kang, 2017) and the present study, 25–50 μM of U0126 inhibited ERK1/2 phosphorylation in the hippocampus by ~50% after 7 day-over infusion. Osmotic pump was placed in a subcutaneous pocket in the interscapular region.

### Electrophysiology

One week after surgery, animals were anesthetized (urethane, 1.5 g/kg, i.p.), removed the infusion kit and osmotic pump, and placed in a stereotaxic frame. A bipolar stimulating electrode was placed in the perforant path (coordinates: 8 mm posterior, 4.4 mm lateral to Bregma, 3.0–3.3 mm depth). A recording electrode was placed in the dentate gyrus and stratum pyramidale of the CA1 region (coordinates: 3.8 mm posterior to Bregma, 2.5 mm lateral to the midline; 2.5 mm (to stratum pyramidale of the CA1 region) or 2.9 mm (to the dentate gyrus) depth). Electrode depths were finally determined by optimizing the evoked response. The reference electrode was placed in the cerebellum. Body temperature was monitored and maintained at 37 ± 0.3°C by thermostat during recording. After establishing a stable baseline for at least 30 min and a control input–output (IO) curve, stimuli were delivered at interstimulus intervals of 30 ms as DC square pulses at 0.5 Hz with pairs of 150-μs constant current stimuli. The stimulus current intensity was adjusted at an intensity yielding 50% of the maximal amplitude of population spike. For sustained stimuli, stimuli were delivered in 9 s long tetanic (30 ms interval) stimulus trains (300 total stimuli). For analysis of the responsiveness of Kv4.3 to 4-aminopyridine (4-AP), animals were injected with saline or 4-AP (20 μM, 1 μl) using a guide-electrode system (C315G-MS303, Plastics One, Roanoke, VA, USA) over a 1-min period using a microinjection pump (1 μl/min, KD Scientific, Hollistone, MA, USA). Signals were recorded with DAM 80 differential amplifier (0.1–3000 Hz bandpass, World Precision Instruments, Sarasota, FL, USA) and data were digitized (20 kHz) and analyzed on LabChart Pro v7 (AD Instruments, Bella Vista, NSW, Australia).

To analyze changes in evoked response, all of the population spike amplitude and field excitatory postsynaptic potential (fEPSP) slope measurements were normalized by the average basement values of the population spike amplitude and fEPSP slope. To measure the efficiency of glutamatergic synaptic transmission in the DG, the excitability ratio was calculated as the population spike amplitude vs. the fEPSP slope in the first response. The ratio of the second population spike amplitude/the first population spike amplitude (population spike amplitude ratio) was also analyzed in order to measure GABA-mediated inhibition in the DG and the CA1 region, respectively (Figure 3A; Andersen et al., 1964, 1971; Lomø, 1971; Buckmaster and Wong, 2002). If multiple population spikes were detected, the first population spike was used for population spike amplitude ratio. After recording, the animal was quickly decapitated for Western blot and immunohistochemistry (Kim et al., 2008, 2016).

**Figure 1:**
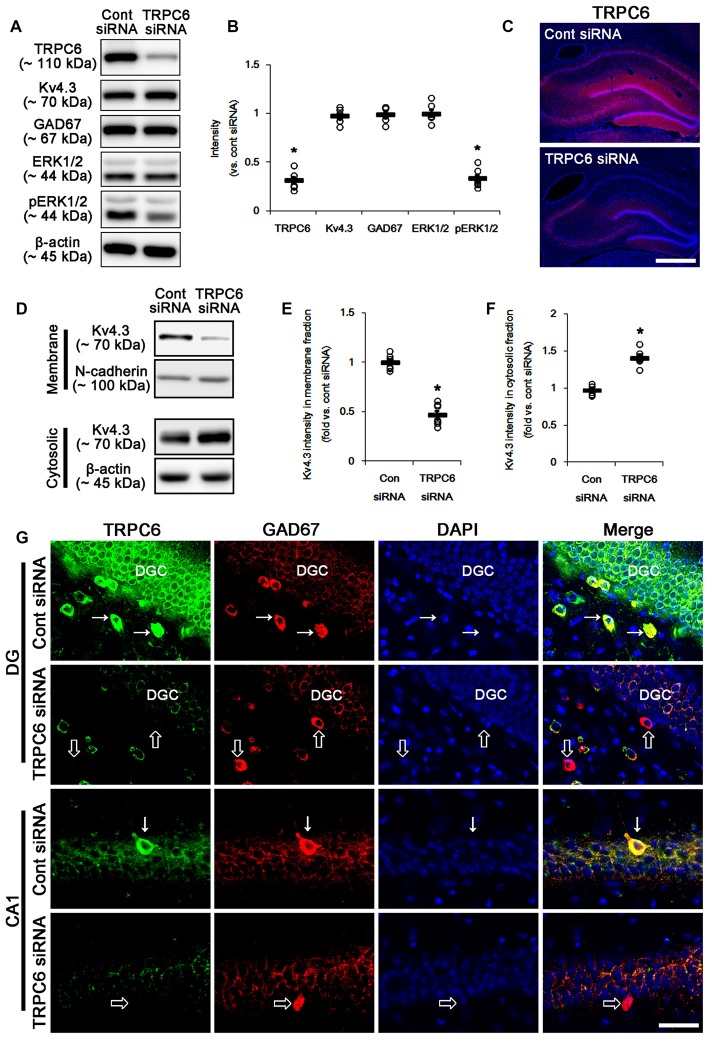
The effect of transient receptor potential channel-6 (TRPC6) knockdown on TRPC6, Kv4.3, GAD67, extracellular signal-regulated kinase 1/2 (ERK1/2) and pERK1/2 levels. **(A)** Representative western blot for TRPC6, Kv4.3, GAD67, ERK1/2 and pERK1/2. TRPC6 siRNA effectively decreases TRPC6 and pERK1/2 levels, but not others. **(B)** Quantification of protein expression and phosphorylation based on the western blots. Open circles indicate each individual value. Horizontal bars indicate mean value. Error bars indicate standard error of the mean (SEM; *p < 0.05 vs. control; *n* = 7, respectively). **(C)** Representative photos demonstrating TRPC6 expression in the hippocampus. As compared to control siRNA, TRPC6 siRNA infusion markedly reduces TRPC6 expression in all hippocampal regions. Bar = 300 μm. **(D)** Representative western data demonstrating the effect of TRPC6 knockdown on Kv4.3 subcellular locations. TRPC6 siRNA reduces membrane translocation of Kv4.3, but increases cytosolic Kv4.3 intensity. **(E)** Quantification of the effect of TRPC6 siRNA on membrane Kv4.3 translocation. Open circles indicate each individual value. Horizontal bars indicate mean value. Error bars indicate SEM (*p < 0.05 vs. control siRNA; *n* = 7, respectively). **(F)** Quantification of the effect of TRPC6 knockdown on cytosolic Kv4.3 intensity. Open circles indicate each individual value. Horizontal bars indicate mean value. Error bars indicate SEM (*p < 0.05 vs. control siRNA; *n* = 7, respectively). **(G)** Representative photos demonstrating TRPC6 expression in γ-aminobutyric acid (GABA)ergic neurons. As compared to control siRNA, TRPC6 expression is significantly reduced in the dentate granule cells (DGC) and GABAergic interneurons without altered GAD67 expression following TRPC6 infusion. Arrows indicate GABAergic neurons showing TRPC6 expression. Open arrows indicate GABAergic neurons showing the absence of TRPC6 expression. Bar = 25 μm.

**Figure 2:**
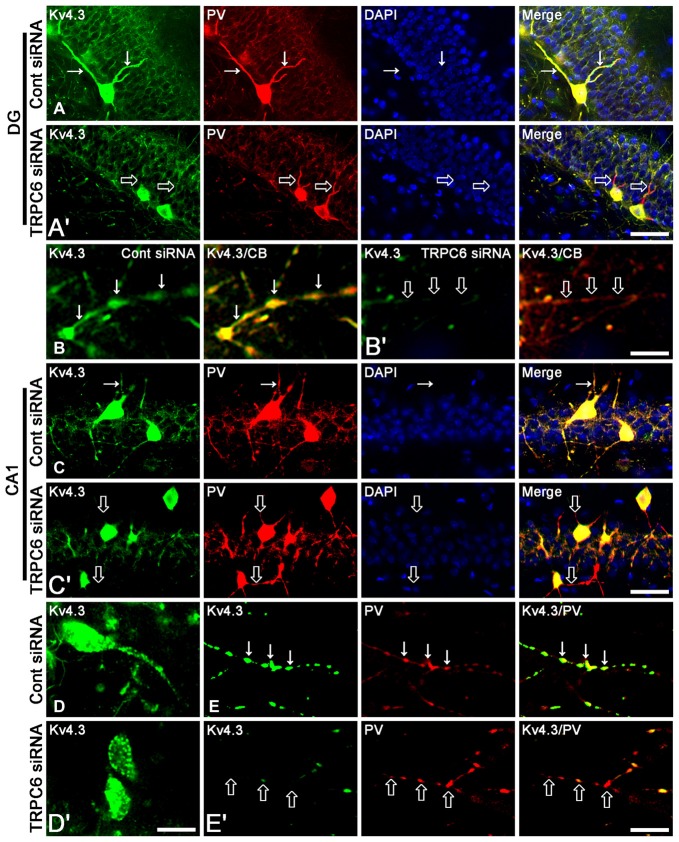
The effect of TRPC6 knockdown on the dendritic Kv4.3 localization. **(A,A′)** Representative photos demonstrating Kv4.3 expression in GABAergic neurons in the DG. Bar = 25 μm. **(B,B′)** Representative photos demonstrating Kv4.3 expression in the CB-positive dendrites of DGC. Bar = 6.25 μm. **(C,C′)** Representative photos demonstrating Kv4.3 expression in GABAergic neurons in the CA1 region. Bar = 25 μm. **(D,D′)** High magnification of Kv4.3 expression in interneurons in the CA1 region. Bar = 12.5 μm. **(E,E′)** Representative photos demonstrating Kv4.3 expression in the PV-positive dendrites of CA1 interneurons. Bar = 6.25 μm. TRPC6 knockdown reduces the dendritic Kv4.2 localization in DGC and interneurons. Arrows indicate the dendritic Kv4.2 localization. Open arrows indicate GABAergic dendrites showing the reduction in Kv4.2 localization.

**Figure 3:**
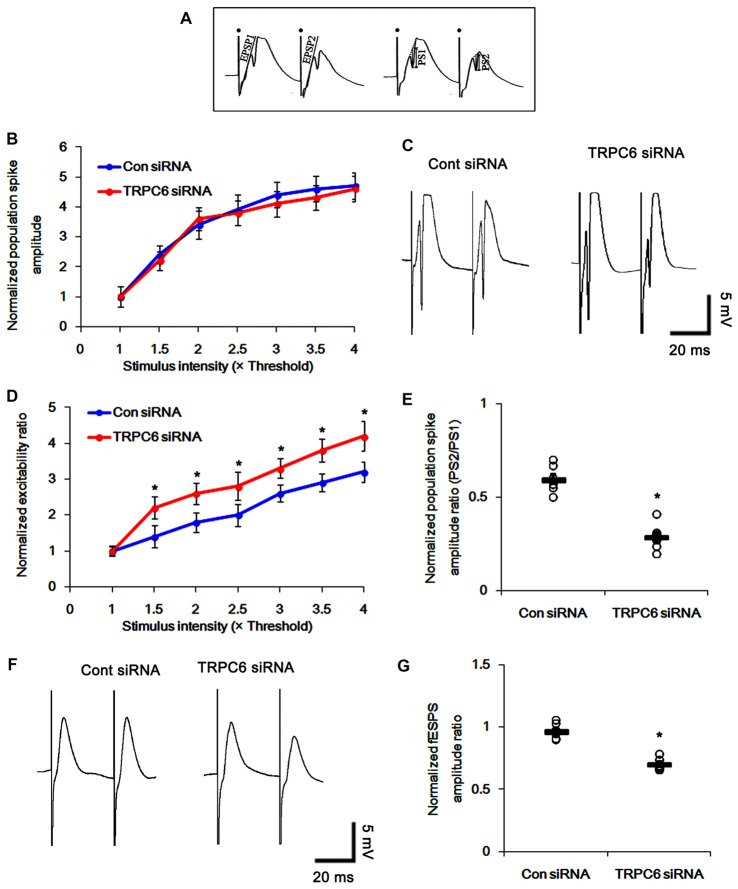
Effect of TRPC6 knockdown on paired-pulse responses in the dentate gyrus and CA1 region.** (A)** Measurement of the field excitatory postsynaptic potential (fEPSP) slope (EPSP) and population spike amplitude (PS). Filled circles indicate stimulus artifacts. **(B)** Input–output (IO) curves for the dentate gyrus of control siRNA- and TRPC6 siRNA-infused animals (mean ± SEM; *n* = 7, respectively). **(C)** Representative traces of paired-pulse responses in the dentate gyrus of control siRNA- and TRPC6 siRNA-infused animals at 30 ms interstimulus interval at stimulus intensity 2× threshold. **(D)** Normalized excitability ratio in the dentate gyrus of control siRNA- and TRPC6 siRNA-infused animals. *p < 0.05 vs. control siRNA-infused animals (mean ± SEM; *n* = 7, respectively). **(E)** Normalized population spike amplitude ratio in the dentate gyrus of control siRNA- and TRPC6 siRNA-infused animals. *p < 0.05 vs. control siRNA-infused animals (mean ± SEM; *n* = 7, respectively). **(F)** Representative traces of paired-pulse responses in the CA1 region of control siRNA- and TRPC6 siRNA-infused animals at 30 ms interstimulus interval at stimulus intensity 2× threshold. **(G)** Normalized fEPSP amplitude ratio in the CA1 region of control siRNA- and TRPC6 siRNA-infused animals. *p < 0.05 vs. control siRNA-infused animals (mean ± SEM; *n* = 7, respectively).

### Subcellular Fraction and Western Blots

The hippocampal was homogenized in lysis buffer. Thereafter, the protein concentration in the supernatant was determined using a Micro BCA Protein Assay Kit (Pierce Chemical, Rockford, IL, USA). To analyze subcellular localization of Kv4.3, we used subcellular Protein Fractionation Kit for Tissues (Thermo Scientific, Waltham, MA, USA), according to the manufacturer’s instructions. Western blotting was performed according to standard procedures. Nitrocellulose transfer membranes were incubated with primary antibodies such as rabbit anti-TRPC6 (1:1000, Millipore, #AB5574), rabbit-anti EKR1/2 (1:1000, Biorbyt, #orb160960), rabbit-anti phospho (p)-ERK1/2 (1:1000, Millipore, #05-797RSP), mouse-anti glutamate decarboxylase 67 (1:1000, GAD67, Millipore, #MAB5406) or rabbit anti-Kv4.3 (1:1000, Alomone labs, #APC-017) antibody. Immunoreactive bands were detected and quantified on ImageQuant LAS4000 system (GE Healthcare, Piscataway, NJ, USA). The rabbit anti-β-actin primary antibody (for cytosolic fraction, 1:6000, Sigma, #A5316) or rabbit anti-N-cadherin (for membrane fraction, 1:1000, Abcam, #ab18203) was used as internal reference.

### Immunohistochemistry

Rats were anesthetized with under urethane anesthesia (1.5 g/kg, i.p.) and perfused through the left ventricle with 200 ml of saline as a vascular rinse followed by a fixative solution containing 4% paraformaldehyde in 0.1 M sodium phosphate buffer (PB), pH 7.4. The brains were removed, postfixed for for 4 h at 4°C, and then cryoprotected by immersion overnight at 4°C in 0.1 M PB containing 30% sucrose. Brains were frozen and serial 30 μm-thick frontal sections were cut with a cryostat. Consecutive sections were contained in six-well plates containing PBS. Free-floating sections were first incubated with 10% normal goat serum (Vector, Burlingame, CA, USA) in PBS for 30 min at room temperature. Sections were then incubated in the mixture of primary antibodies at room temperature for overnight in a solution containing rabbit anti-TRPC6 antibody (1:100, Millipore), rabbit anti-Kv4.3 antibody (1:100, Alomone labs), rabbit anti-Kv4.3 (1:100, Alomone labs)/mouse anti-GAD67 (1:250) or rabbit anti-Kv4.3 (1:100, Alomone labs)/mouse anti-parvalbumin (anti-PV, an interneuron marker, 1:1000, Millipore, #MAB1572) or rabbit anti-Kv4.3 (1:100, Alomone labs)/mouse anti-calbindin D-28k antisera (CB, a granule cell marker, 1:200, SWANT, #300) in PBS containing 0.3% triton X-100. After washing in PBS, sections were incubated for 1 h in a FITC- or Cy3-conjugated secondary antiserum. For nuclei counterstaining, Vectashield mounting medium with DAPI (Vector, Burlingame, CA, USA) was used as a mountant. The antibody that was preincubated with 1 μg of purified peptide (for TRPC6 and Kv4.3) or mouse pre-immune serum (Sigma, #M5905; for other antibodies) was used as for negative control. As the result of negative control test, no immunoreactive structure was observed. Images were captured using an AxioImage M2 microscope or a confocal laser-scanning microscope (LSM 710, Carl Zeiss Inc., Oberkocken, Germany).

### Quantification of Data and Statistical Analysis

A single data point obtained from each animal was used for analysis. All parameters were tested for normal distribution and homogeneity of variances. For variables that fulfilled both assumptions, statistical differences were determined using Mann–Whitney test or ANOVA to determine statistical significance. Bonferroni’s test was used for *post hoc* comparisons. Values are presented as mean ± standard error of the mean (SEM). A *p*-value below 0.05 was considered statistically significant.

## Results

### TRPC6 Knockdown Reduces the Membrane Kv4.3 Translocation and its Dendritic Localization

Figure 1 shows that TRPC6 siRNA resulted in an approximate 70% reduction of TRPC6 protein level and ERK1/2 phosphorylation in the total extract obtained from hippocampus, as compared with control (non-targeting) siRNA (*p* < 0.05, respectively; Figures 1A,B and Supplementary Figure S1), although it did not affect Kv4.3, GAD67 and ERK1/2 expression levels. Consistent with our previous studies (Kim et al., 2013; Kim and Kang, 2015; Ko and Kang, 2017), TRPC6 expression was predominantly observed in DGC and the molecular layer of the dentate gyrus (Figure 1C). TRPC6 knockdown decreased TRPC6 expression in the hippocampus (*p* < 0.05 vs. control siRNA, Figure 1C). Furthermore, TRPC6 knockdown reduced the membrane localization of Kv4.3, but increased the cytosolic Kv4.3 intensity (*p* < 0.05 vs. control siRNA, Figures 1D–F and Supplementary Figure S1). TRPC6 expression was also detected in GABAergic (GAD67-positive) interneurons in the hilus of the dentate gyrus and the CA1 region (Figure 1G). TRPC6 siRNA effectively reduced TRPC6 expression in DGC and interneurons, although it could not influence on GAD67 expression (Figure 1G). Furthermore, TRPC6 knockdown reduced Kv4.3 clusters in the dendrites of CB-positive DGC and PV-positive interneurons (Figures 2A–E). Taken together, our findings indicate TRPC6 knockdown may abolish the dendritic and membrane Kv4.3 localization of in DGC and interneurons.

### TRPC6 Knockdown Increases Neuronal Excitability and Paired-Pulse Inhibition in Response to a Paired-Pulse Stimulation

To determine whether TRPC6 is involved in neuronal excitability, we tested the effect of TRPC6 siRNA infusion on the evoked responses of the DG and CA1 region *in vivo*. TRPC6 knockdown did not affect the IO curve in the DG (Figure 3B), indicating no effect of TRPC6 knockdown on the basal neurotransmission. Consistent with our previous study (Kim and Kang, 2015), TRPC6 knockdown increased the DG excitability ratio and a paired-pulse inhibition at 30-ms interstimulus interval (*p* < 0.05 vs. control siRNA, Figures 3C–E). In contrast to the DG, the perforant path stimulation evoked only fEPSP in the CA1 region. TRPC6 knockdown reduced fEPSP amplitude ratio in the CA1 region (*p* < 0.05 vs. control siRNA, Figures 3F,G). Excitability ratio is an index of synaptic efficacy, DGC excitability and the threshold of epileptiform discharges. In addition, paired-pulse response represents functional changes of GABAergic inhibition (Kim and Kang, 2015). Therefore, our findings indicate that the silencing of TRPC6 increases excitability and inhibitory transmission in the DGC and the CA1 region.

### TRPC6 Knockdown Impairs GABAergic Inhibition in the DG and the CA1 Region during Sustained Repetitive Stimuli

**Figure 4:**
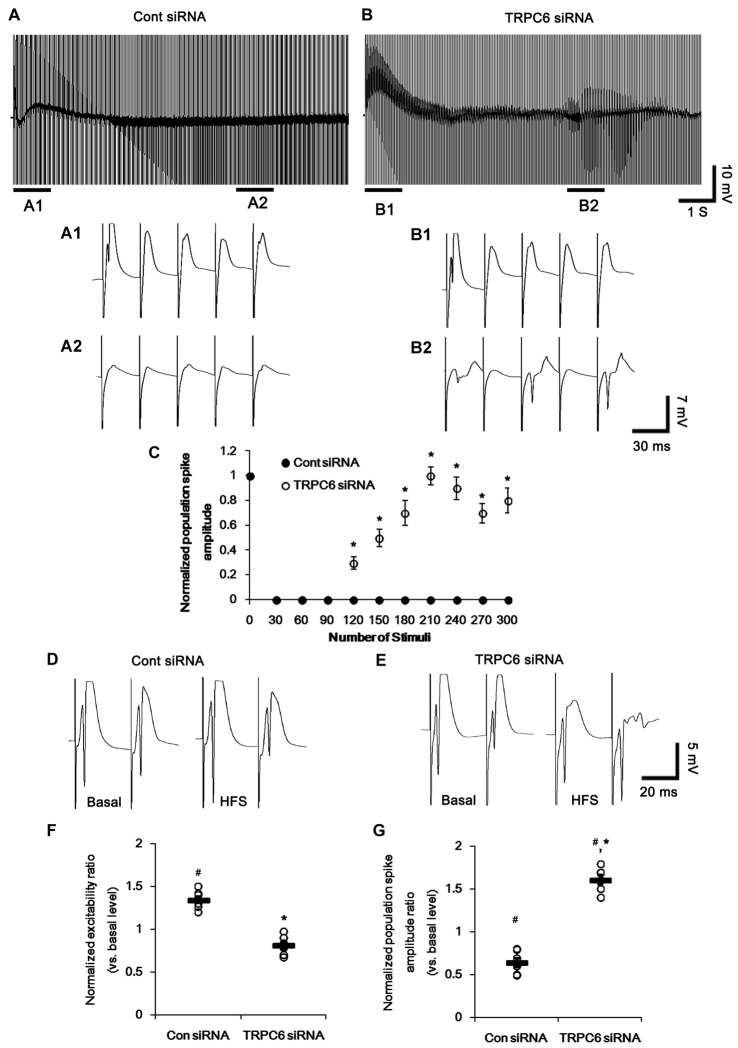
Effect of high-frequency stimulation (HFS) on evoked responses in the dentate gyrus. **(A,B)** Representative traces of the evoked potentials during HFS in the dentate gyrus of control siRNA- **(A)** and TRPC6 siRNA-infused animals **(B)**. Bars **(A1,2,B1,2)** indicate the time window expanded in the below panels. **(C)** Normalized population spike amplitude in the dentate gyrus of control siRNA- and TRPC6 siRNA-infused animals. *p < 0.05 vs. control siRNA-infused animals (mean ± SEM; *n* = 7, respectively). **(D,E)** Representative traces of paired-pulse responses in the dentate gyrus of control siRNA- and TRPC6 siRNA-infused animals at 30 ms interstimulus interval at stimulus intensity 2× threshold in basal level (left) and post-stimulation (right). **(F)** Normalized excitability ratio in the dentate gyrus of control siRNA- and TRPC6 siRNA-infused animals. ^#^,*p < 0.05 vs. basal level and control siRNA-infused animals, respectively (mean ± SEM; *n* = 7, respectively). **(G)** Normalized population spike amplitude ratio in the dentate gyrus of control siRNA- and TRPC6 siRNA-infused animals. ^#^,*p < 0.05 vs. basal level and control siRNA-infused animals, respectively (mean ± SEM; *n* = 7, respectively).

In the present study, TRPC6 siRNA reduced Kv4.3 clusters in dendrites of DGC and interneurons (Figure 2). Thus, it is likely that TRPC6 may play a critical role in subcellular Kv4.3 localization. Since Kv4.3 promotes stronger inhibitory control of firing during sustained activity (Bourdeau et al., 2011), we investigated in more detail the effect of TRPC6 knockdown on the network behaviors of the DG and the CA1 region following repetitive stimulus train (300 stimuli in 9 S) of the perforant path. Control siRNA-infused animals showed steady fEPSP depression in response to repetitive stimulation of perforant path (Figures 4A,A1,A2,C). However, TRPC6 siRNA-infused animals showed population spike generation during repetitive stimulation (*p* < 0.05 vs. control siRNA; Figures 4B,B1,B2,C). No stimulus-induced afterdischarge was observed in both groups (data not shown).

Following high-frequency stimulation (HFS), control siRNA-infused animals showed the increases in excitability ratio (1.34-fold of basal level) and paired-pulse inhibition (0.64-fold of basal level) in the DG (*p* < 0.05 vs. basal level, Figures 4D,F,G). TRPC6 siRNA-infused animals revealed the elevated population spike amplitude ratio (1.61-fold of basal level) and resulted in multiple population spikes in the second pulse without changed excitability ratio (*p* < 0.05 vs. control siRNA; Figures 4E–G).

**Figure 5:**
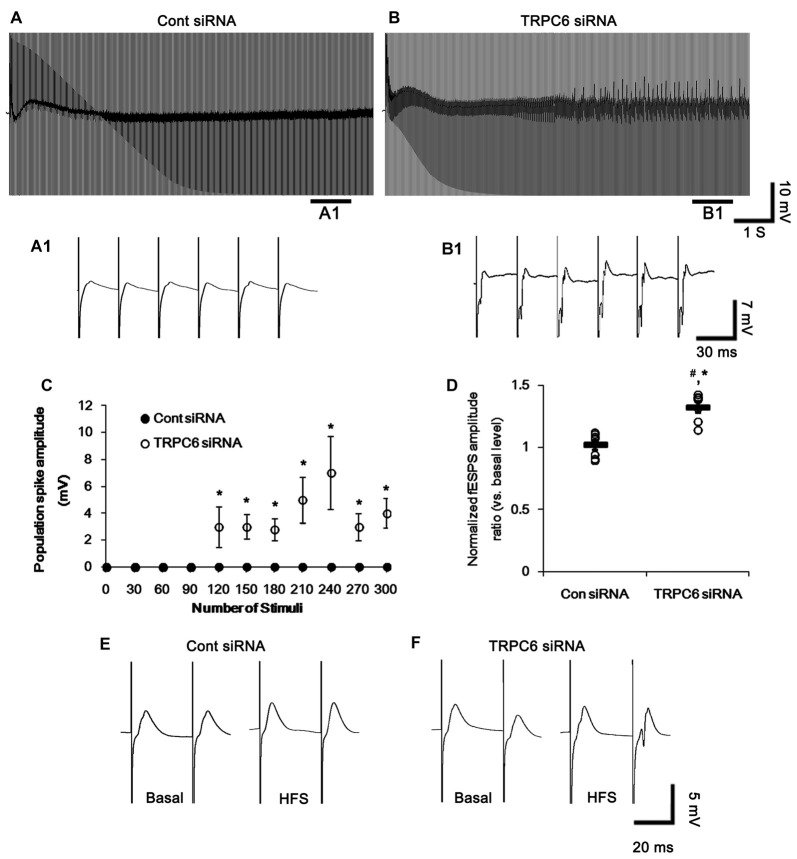
Effect of HFS on evoked responses in the CA1 region. **(A,B)** Representative traces of the evoked potentials during HFS in the CA1 region of control siRNA- **(A)** and TRPC6 siRNA-infused animals **(B)**. Bars **(A1,B1)** indicate the time window expanded in the below panels. **(C)** Population spike amplitude in the CA1 region of control siRNA- and TRPC6 siRNA-infused animals. *p < 0.05 vs. control siRNA-infused animals (mean ± SEM; *n* = 7, respectively). **(D)** Normalized fEPSP amplitude ratio in the CA1 region of control siRNA- and TRPC6 siRNA-infused animals. ^#^,*p < 0.05 vs. basal level and control siRNA-infused animals, respectively (mean ± SEM; *n* = 7, respectively). **(E,F)** Representative traces of paired-pulse responses in the CA1 region of control siRNA- and TRPC6 siRNA-infused animals at 30 ms interstimulus interval at stimulus intensity 2× threshold in basal level (left) and post-stimulation (right).

In the CA1 region, HFS provoked a population spike in TRPC6-infused animals (*p* < 0.05 vs. control siRNA; Figures 5A,A1,B,B1,C). Following HFS, fEPSP amplitude ratio in response to paired-pulse stimulation was unaltered in control siRNA-infused animals, but increased in TRPC6-infused animals (*p* < 0.05 vs. control siRNA; Figures 5D–F). Furthermore, TRPC6 siRNA-infused animals showed a population spike in the CA1 region in response to the second perforant path stimulus (Figures 5D–F). Since population spikes are invariably coupled to synchronous population bursts of GABAergic interneurons (Ylinen et al., 1995), our findings indicate that TRPC6 knockdown-mediated Kv4.3 translocation may decrease GABAergic inhibition in response to sustained repetitive stimuli.

### TRPC6 Knockdown Decreases the Responsiveness to 4-AP

**Figure 6:**
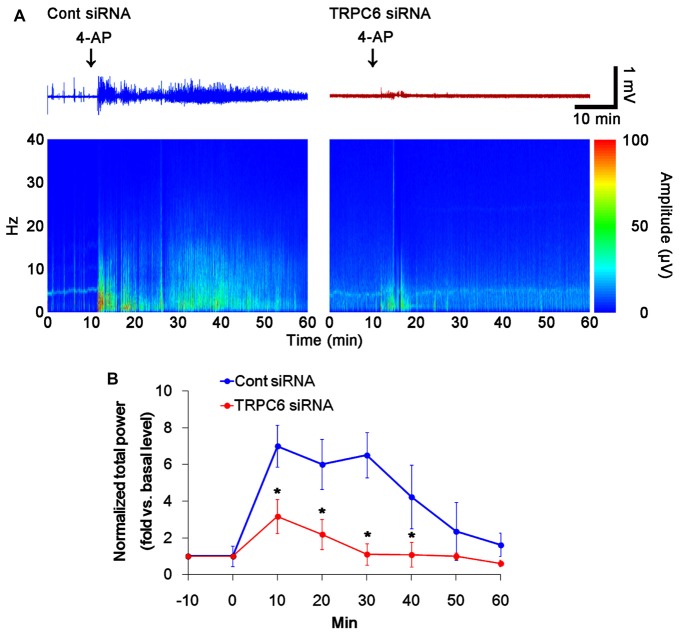
Effect of TRPC6 knockdown on the responsiveness to 4-AP. As compared to control siRNA-infused animals, TRPC6 siRNA-infused animals reveal lower responsiveness to 4-AP. **(A)** Representative EEG traces and frequency-power spectral temporal maps in response to 4-AP. **(B)** Quantification of total EEG power in response to 4-AP (mean ± SEM; *p < 0.05 vs. control siRNA; *n* = 7, respectively).

Similar to TRPC6 knockdown in the present study, 4-AP (an A-type K^+^ channel inhibitor) increases field potentials, accompanied by synchronously giant inhibitory postsynaptic potential in DGC and CA3 pyramidal cells (Müller and Misgeld, 1990, 1991; Otis and Mody, 1992). Thus, it is likely that TRPC6 knockdown would influence on the responsiveness to 4-AP, if altered Kv4.3 translocation induced by TRPC6 knockdown affected the electrophysiological properties of the DG and the CA1 region. To elucidate this hypothesis, we investigated the effect of TRPC6 knockdown on the responsiveness to 4-AP (20 μM). In control siRNA-infused animals, 4-AP significantly elevated total power as compared to basal level. However, the 4-AP infusion was less effective in TRPC6 siRNA-infused animals (*p* < 0.05 vs. control siRNA, Figures 6A,B). With respect to reduced efficacy of 4-AP in TRPC6 knockdown animals in the present study, our findings provide the possibility that TRPC6 may play important roles in the regulation of subcellular Kv4.3 localization.

### Blockade of ERK1/2 Reduces Excitability Ratio, but Generates Multiple Population Spikes in the DG

**Figure 7:**
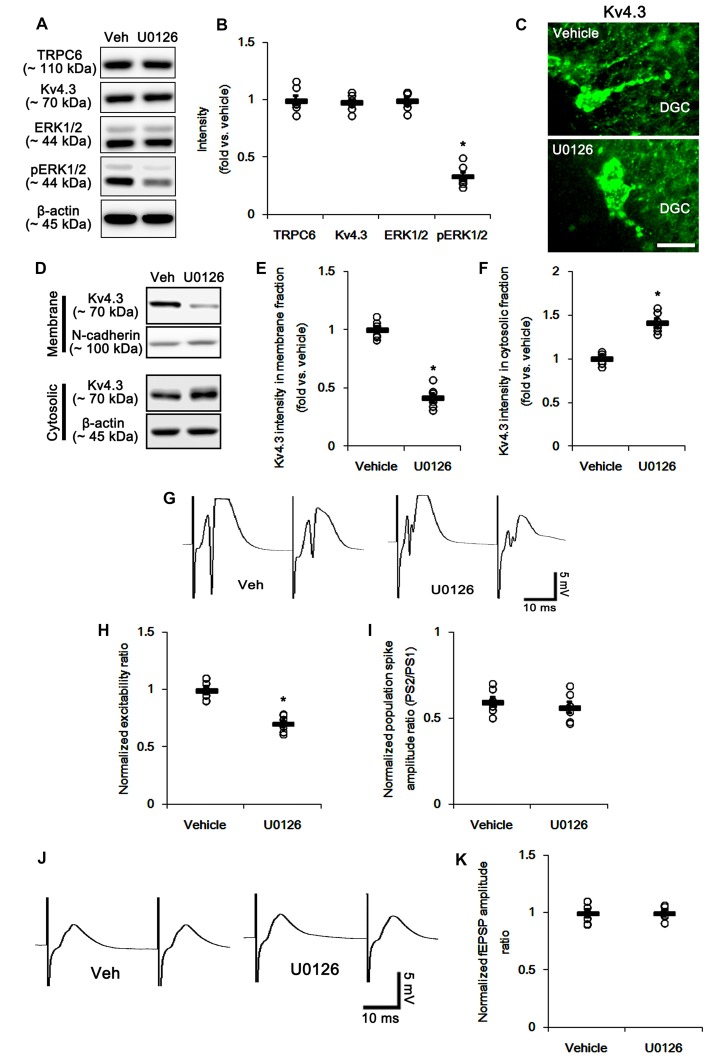
The effect of U0126 on the hippocampal properties. **(A)** Representative western blot for TRPC6, Kv4.3, ERK1/2 and pERK1/2. U0126 effectively decreases pERK1/2 levels, but not others. **(B)** Quantification of protein expression and phosphorylation based on the western blots. Open circles indicate each individual value. Horizontal bars indicate mean value. Error bars indicate SEM (*p < 0.05 vs. vehicle; *n* = 7, respectively). **(C)** Representative photos demonstrating Kv4.3 expression in the hilar interneurons. As compared to control vehicle, U0126 markedly reduces the dendritic Kv4.3 localization. Bar = 12.5 μm. **(D)** Representative western data demonstrating the effect of U0126 on Kv4.3 subcellular locations. U0126 reduces membrane translocation of Kv4.3, but increases cytosolic Kv4.3 intensity. **(E)** Quantification of the effect of U0126 on membrane Kv4.3 translocation. Open circles indicate each individual value. Horizontal bars indicate mean value. Error bars indicate SEM (*p < 0.05 vs. vehicle; *n* = 7, respectively). **(F)** Quantification of the effect of U0126 on cytosolic Kv4.3 intensity. Open circles indicate each individual value. Horizontal bars indicate mean value. Error bars indicate SEM (*p < 0.05 vs. vehicle; *n* = 7, respectively). **(G)** Representative traces of paired-pulse responses in the dentate gyrus of vehicle- and U0126-treated animals at 30 ms interstimulus interval at stimulus intensity 2× threshold. **(H)** Normalized excitability ratio in the dentate gyrus of vehicle- and U0126-treated animals. *p < 0.05 vs. vehicle-infused animals (mean ± SEM; *n* = 7, respectively). **(I)** Normalized population spike amplitude ratio in the dentate gyrus of vehicle- and U0126-treated animals (mean ± SEM; *n* = 7, respectively). **(J)** Representative traces of paired-pulse responses in the CA1 region of vehicle- and U0126-treated animals at 30 ms interstimulus interval at stimulus intensity 2× threshold. **(K)** Normalized fEPSP amplitude ratio in the CA1 region of vehicle- and U0126-treated animals (mean ± SEM; *n* = 7, respectively).

Next, we investigated how TRPC6 knockdown regulates subcellular Kv4.3 localization. Consistent with our previous study (Ko and Kang, 2017), the present study revealed that TRPC6 siRNA inhibits ERK1/2 phosphorylation in the hippocampus (Figures 1A,B). Furthermore, ERK1/2 plays a key role in the subcellular Kv4.3 localization (Setién et al., 2013). Therefore, we explored whether TRPC6 knockdown-mediated ERK1/2 inhibition is involved in the alterations in neuronal excitability, paired-pulse responses and subcellular Kv4.3 localization in the hippocampus. U0126, an ERK1/2 inhibitor, reduced ERK1/2 phosphorylation, but not expressions of ERK1/2, TRPC6 and Kv4.3 (Figures 7A,B and Supplementary Figure S2). U0126 decreased Kv4.3 clusters in the dendrites of interneurons (Figure 7C). U0126 also reduced Kv4.3 intensity in the membrane fraction, but increased it in the cytosolic fraction (*p* < 0.05 vs. vehicle, Figures 7D–F and Supplementary Figure S2). U0126 did not affect population spike amplitude ratio, but decreased excitability ratio. However, U0126 generated noteworthy multiple population spikes in both the first and the second pulse in the DG. U0126 did not affect paired-pulse responses in the CA1 regions of both groups (Figures 7F–K). Since the reduced excitability ratio represent the decreases in synaptic efficacy and neuronal excitability (Kim and Kang, 2015), our findings reveal that U0126 may diminish synaptic efficacy and neuronal excitability of DGC. However, multiple population spikes demonstrate the synchrony and the “more excitable” properties of DG (Uruno et al., 1994). Therefore, our findings indicate that U0126 may generate synchronous population bursts in the DGC via diminishing membrane Kv4.3 translocation.

**Figure 8:**
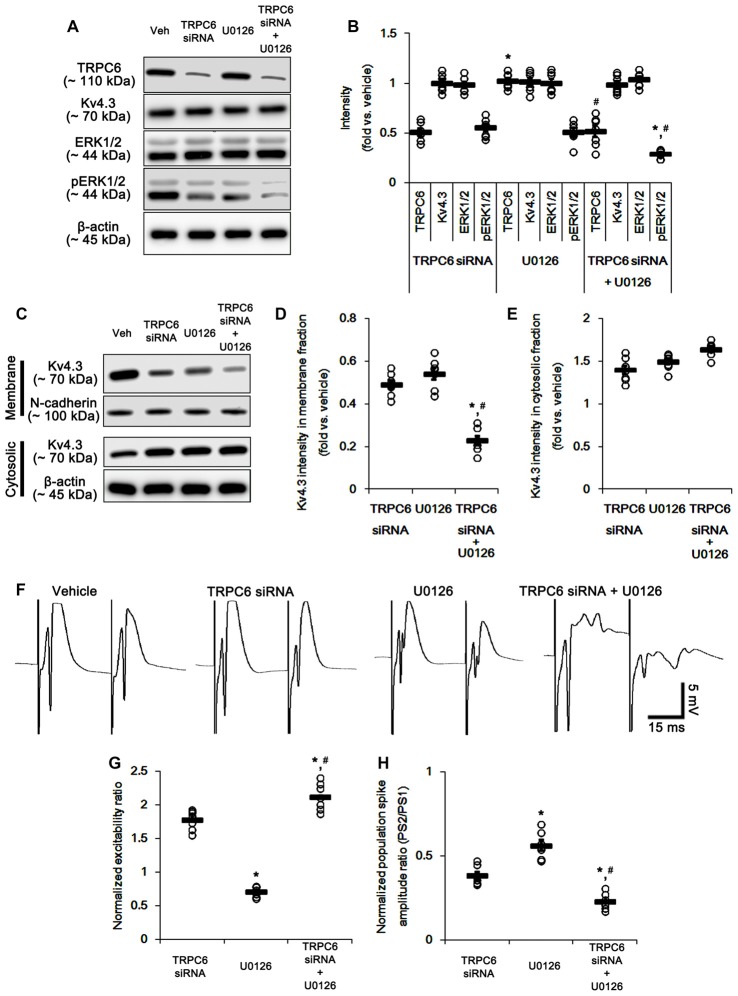
The effect of co-treatment of TRPC6 and U0126 on the hippocampal properties. **(A)** Representative western blot for TRPC6, Kv4.3, ERK1/2 and pERK1/2. Co-treatment effectively decreases pERK1/2 levels and TRPC6 expression, but not others. **(B)** Quantification of protein expression and phosphorylation based on the western blots. Open circles indicate each individual value. Horizontal bars indicate mean value. Error bars indicate SEM (*,^#^*p* < 0.05 vs. TRPC6 and U0126, respectively; *n* = 7, respectively). **(C)** Representative western data demonstrating the effect of co-treatment of TRPC6 and U0126 on Kv4.3 subcellular locations. As compared to TRPC6 and U0126, co-treatment more reduces membrane translocation of Kv4.3, but not cytosolic Kv4.3 intensity. **(D)** Quantification of the effect of co-treatment of TRPC6 and U0126 on membrane Kv4.3 translocation. Open circles indicate each individual value. Horizontal bars indicate mean value. Error bars indicate SEM (*,^#^*p* < 0.05 vs. TRPC6 and U0126, respectively; *n* = 7, respectively). **(E)** Quantification of the effect of co-treatment of TRPC6 and U0126 on cytosolic Kv4.3 intensity. Open circles indicate each individual value. Horizontal bars indicate mean value. Error bars indicate SEM (*n* = 7, respectively). **(F)** Representative traces of paired-pulse responses in the dentate gyrus of vehicle-, TRPC6 siRNA-, U0126- and co-treatment of TRPC6 and U0126-treated animals at 30 ms interstimulus interval at stimulus intensity 2× threshold. **(G)** Normalized excitability ratio in the dentate gyrus of TRPC6 siRNA-, U0126- and co-treatment of TRPC6 and U0126-treated animals. Error bars indicate SEM (*,^#^*p* < 0.05 vs. TRPC6 and U0126, respectively; *n* = 7, respectively). **(H)** Normalized population spike amplitude ratio in the dentate gyrus of vehicle-, TRPC6 siRNA-, U0126- and co-treatment of TRPC6 and U0126-treated animals. Error bars indicate SEM (*,^#^*p* < 0.05 vs. TRPC6 and U0126, respectively; *n* = 7, respectively).

To detail the role of TRPC6-mediated ERK1/2 activation in neuronal excitability, we applied co-treatment of TRPC6 siRNA and U0126, and compared its effect to those of TRPC6 and U0126. Similar to TRPC6 siRNA, co-treatment of TRPC6 siRNA and U0126 significantly reduced TRPC6 expression (Figures 8A,B and Supplementary Figure S3). The effect of TRPC6 siRNA on ERK1/2 phosphorylation, Kv4.3 and subcellular Kv4.3 localization was similar to that of U0126 (Figures 8A–E and Supplementary Figure S3). Co-treatment of TRPC6 siRNA and U0126 decreased ERK1/2 phosphorylation and membrane Kv4.3 localization more than TRPC6 and U0126 (*p* < 0.05 vs. TRPC6 and U0126; Figures 8A–E and Supplementary Figure S3). Co-treatment slightly increased Kv4.3 level in cytosolic fraction, but it was not statistically significant (Figures 8C–E and Supplementary Figure S3). As compared to TRPC6 knockdown or U0126, co-treatment of TRPC6 and U0126 more increased excitability ratio and generated more multiple population spikes, accompanied by enhanced paired-pulse inhibition (*p* < 0.05 vs. TRPC6 and U0126; Figures 8F–H). Taken together, our findings suggest TRPC6-mediated ERK1/2 activation may inhibit synchronous population bursts in the DGC via regulating membrane Kv4.3 localization.

### TRPC6 Activation Reduces Excitability Ratio without Changed Paired-Pulse Response in the DG

**Figure 9:**
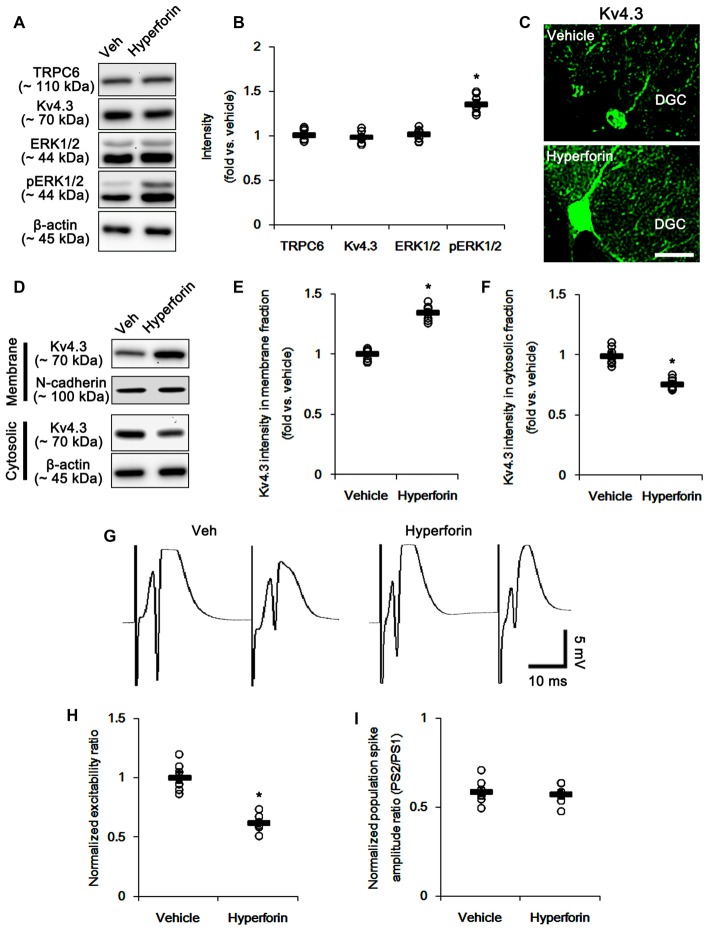
The effect of hyperforin on the hippocampal properties. **(A)** Representative western blot for TRPC6, Kv4.3, ERK1/2 and pERK1/2. hyperforin effectively increases pERK1/2 levels, but not others. **(B)** Quantification of protein expression and phosphorylation based on the western blots. Open circles indicate each individual value. Horizontal bars indicate mean value. Error bars indicate SEM (*p < 0.05 vs. vehicle; *n* = 7, respectively). **(C)** Representative photos demonstrating Kv4.3 expression in the hilar interneurons. As compared to control vehicle, hyperforin markedly increases the dendritic Kv4.3 localization. Bar = 12.5 μm. **(D)** Representative western data demonstrating the effect of hyperforin on Kv4.3 subcellular locations. Hyperforin increases membrane translocation of Kv4.3, but reduces cytosolic Kv4.3 intensity. **(E)** Quantification of the effect of hyperforin on membrane Kv4.3 translocation. Open circles indicate each individual value. Horizontal bars indicate mean value. Error bars indicate SEM (*p < 0.05 vs. vehicle; *n* = 7, respectively). **(F)** Quantification of the effect of hyperforin on cytosolic Kv4.3 intensity. Open circles indicate each individual value. Horizontal bars indicate mean value. Error bars indicate SEM (*p < 0.05 vs. vehicle; *n* = 7, respectively). **(G)** Representative traces of paired-pulse responses in the dentate gyrus of vehicle- and hyperforin-treated animals at 30 ms interstimulus interval at stimulus intensity 2× threshold. **(H)** Normalized excitability ratio in the dentate gyrus of vehicle- and hyperforin-treated animals. *p < 0.05 vs. vehicle-infused animals (mean ± SEM; *n* = 7, respectively). **(I)** Normalized population spike amplitude ratio in the dentate gyrus of vehicle- and hyperforin-treated animals (mean ± SEM; *n* = 7, respectively).

The remaining question is whether TRPC6 activation directly increases membrane Kv4.3 localization via enhanced ERK1/2 activity. Since hyperforin, a TRPC6 activator (Leuner et al., 2007), increases ERK1/2 activity in hippocampal neurons (Heiser et al., 2013), we explored the effects of hyperforin on evoked potentials and subcellular Kv4.3 localization. Consistent with a previous study (Heiser et al., 2013), hyperforin increased ERK1/2 phosphorylation (*p* < 0.05 vs. vehicle; Figures 9A,B and Supplementary Figure S4) without changed TRPC6 and Kv4.3 expressions. Furthermore, hyperforin elevated the dendritic and membrane Kv4.3 localizations (*p* < 0.05 vs. vehicle; Figures 9C–F and Supplementary Figure S4). Hyperforin also reduced excitability ratio due to the decreased population spike amplitudes (*p* < 0.05 vs. vehicle; Figures 9G–I), while it did not affect paired-pulse inhibitions. These findings suggest that TRPC6-mediated ERK1/2 activation may play an important role in the maintenances of neuronal excitability and membrane Kv4.3 localization in the DG.

## Discussion

Recently, we have reported that down-regulation of TRPC6 expression increases the susceptibility to ictogenic stimuli and neuronal excitability combined with a high level of synchrony of neurons. Paradoxically, TRPC6 knockdown also enhances GABAergic inhibitory input to DGC (Kim and Kang, 2015). In the present study, therefore, we accessed the underlying mechanisms of the regulation of TRPC6-meidated neuronal excitability and the entrainment of GABAergic interneuron activity, which have been less understood.

Consistent with our previous study (Kim and Kang, 2015), the present study demonstrates that TRPC6 knockdown increased paired-pulse inhibition in response to single paired-pulse stimulus. Regarding to the inhibitory role of TRPC6 in NMDA receptor activation (Li et al., 2012; Shen et al., 2013), these findings indicate that TRPC6 knockdown may increase GABAergic inhibition by enhancing the responsiveness of NMDA receptor on interneurons. This is because activation of NMDA receptor on interneurons increases the inhibitory input in principal neurons (Sharp et al., 2001; Krystal et al., 2003). In the present study, we also found that TRPC6 siRNA reduced membrane Kv4.3 translocation and its dendritic localization in interneurons. The blockade of A-type K^+^ channels induces burst discharges in interneurons independent of NMDA and amino-3-hydroxy-5-methyl-isoxazole propionic acid (AMPA) receptor-mediated excitatory neurotransmission, and consequently occurs large inhibitory postsynaptic potentials accompanied by large field potentials in DGC and CA3 pyramidal cells (Müller and Misgeld, 1990, 1991; Otis and Mody, 1992). With respect to these previous studies, our findings provide a possibility that TRPC6 knockdown may enhance the intact steady-state activation of interneurons via reducing membrane Kv4.3 localization on GABAergic interneurons.

Unlike single paired-pulse stimulus, the present data showed that TRPC6 siRNA reduced GABAergic inhibitions onto the DGC and CA1 pyramidal cells during and after HFS, concomitant with decreases in membrane Kv4.3 translocation and its dendritic localization in interneurons. These findings indicate that TRPC6 may be involved in the maintenance of GABAergic inhibition in response to fast, repetitive and long-term stimuli. Prominent GABAergic innervations produce long-lasting inhibitory postsynaptic potentials that reduce afterdischarges and prevent generation of burst discharges (Andersen et al., 1971; McNaughton and Barnes, 1977). Thus, GABAergic neurons have the fast-spiking capability that is important to maintain the responsiveness to an adaptation to repetitive spikes (Cammarota et al., 2013; Elgueta et al., 2015). In particular, Kv4.3 underlies A-type K^+^ currents that modulates the recovery from inactivation and the repetitive firing in interneurons (Bourdeau et al., 2007, 2011). Indeed, dysfunction of Kv4.3 impairs inhibitory control of firing in during sustained activity (Bourdeau et al., 2011). Therefore, the reduction in GABAergic inhibitions on the DGC and CA1 pyramidal cells in response to HFS indicate that TRPC6 knockdown may impair recovery from inactivation of interneurons due to reduced membrane Kv4.3 translocation. Taken together, our findings suggest that TRPC6 may play an important role in the maintenance of the fast-spiking activity in interneurons by facilitating the membrane Kv4.3 translocation.

In the present study, TRPC6 knockdown increased the excitability ratio in DGC accompanied by reduced membrane Kv4.3 localization and the responsiveness to 4-AP. Since the excitability ratio is not relevant to paired-pulse inhibition of DGC (Varaschin et al., 2014), the increased excitability ratio may be an indicative of the changed intrinsic electrical properties of DGC resulting in the lowered action potential threshold independent of GABAergic inhibition. Furthermore, the reduction in Kv4.3 expression decreases the sensitivity to 4-AP (Gao et al., 2010). Given the role of A-type K^+^ channels in the backward spread of action potentials and integration of EPSPs (Hoffman et al., 1997; Kim et al., 2007), the down-regulation of the dendritic Kv4.3 localization in DGC may be one of the mechanisms of increased excitability ratio induced by TRPC6 knockdown. Furthermore, Kv4.3 is involved in Ca^2+^-dependent regulation of A-type K^+^ currents in neurons (Rhodes et al., 2004; Bourdeau et al., 2007; Menegola et al., 2008). Based on these previous reports, it is likely that TRPC6 may participate in Kv4.3-mediated A-type K^+^ current generation via regulating membrane Kv4.3 translocation in DGC, although we could not access the Kv4.3 functionality by a direct electrophysiological approach.

On the other hand, some A-type K^+^ channel functions and their localizations are modified by phosphorylation. For example, Kv4.2 phosphorylation results in a decrease in the number of channels on the cell surface, which profoundly increases action potential amplitude and dendritic excitability (Gupte et al., 2016). Unlike Kv4.2, the membrane Kv4.3 translocation is increased by ERK1/2 activation (Setién et al., 2013). Consistent with our previous study (Ko and Kang, 2017), the present study exhibits that TRPC6 knockdown reduced ERK1/2 activity in the hippocampus. Furthermore, U0126 abolished the membrane Kv4.3 translocation and its dendritic localization. ERK1/2 inhibition reduces the efficacy of 4-AP in the dentate gyrus (Merlo et al., 2004), which is relevant to Kv4.3 expression (Gao et al., 2010). Therefore, our findings indicate that TRPC6-mediated ERK1/2 activation may regulate the subcellular Kv4.3 localization, similar to large-conductance Ca^2+^-activated K^+^ channels (BK_Ca_ channels, Kim et al., 2009).

The role of ERK1/2 in the regulation of neuronal activity has been still controversial. EKR1/2 inhibitors attenuate epileptiform discharges (Glazova et al., 2015; Ko and Kang, 2017). In addition, persistent ERK1/2 activation results in spontaneous seizures (Nateri et al., 2007). ERK1/2 inhibition also rescues 4-AP-induced epileptiform activity without any change in the amplitude and latency of the field potential responses to electrical stimuli delivered in the dentate gyrus (Merlo et al., 2004). However, U0126 blocks the suppressive effects of erythropoietin and insulin on neuronal hyperexcitability induced by kainite- or NMDA receptor activation (O’Malley et al., 2003; Zheng et al., 2013). Furthermore, EKR1/2 inhibition accelerates Kv4.3 inactivation by a direct action on channel gating (Yuan et al., 2006). Considering these previous studies, it is likely that ERK1/2 may be a secondary regulatory modulator of neuronal excitability in response to various channel activities, which may show divergent effects. Unexpectedly, the present study revealed that U0126 generated multiple population spikes in the DG and reduced excitability ratio without changed population spike amplitude ratio, which were adverse to TRPC6 knockdown. In addition, the present study demonstrate that co-treatment of TRPC6 and U0126 more increased excitability ratio and generated more multiple population spikes, accompanied by enhanced paired-pulse inhibition. Since excitability ratio, so-called fEPSP slope-population spike amplitude (E-S) coupling, is an index of synaptic efficacy and neuronal excitability (Kim and Kang, 2015) and multiple population spikes represent the DGC synchrony (Uruno et al., 1994), our findings suggest that TRPC6 knockdown-mediated ERK1/2 inactivation may be responsible for multiple synchronous population bursts as well as neuronal excitability in DGC via reduced membrane Kv4.3 localization, although cause-effect relationship between ERK1/2 activity and neuronal excitability remains to be verified in further investigations.

In the present study, we also found that hyperforin increased ERK1/2 phosphorylation and membrane Kv4.3 localization. In our previous study (Lee et al., 2014), the concentration of hyperforin applied in the present study (6 μM) cannot affect the seizure susceptibility in response to pilocarpine. In the present study, however, hyperforin reduced excitability ratio due to the decreased population spike amplitudes. Although hyperforin elevated the dendritic Kv4.3 localizations in interneurons, there was no difference in paired-pulse inhibition between vehicle and hyperforin. These findings are consistent with a previous report (Langosch et al., 2002) demonstrating the effects of hyperforin on evoked potentials: The higher concentration of hyperforin (10 μM) reduces the population spike amplitudes without changed paired-pulse responses, while the lower concentration (1 μM) slightly increases the population spike amplitudes. Furthermore, our findings indicate that hyperforin may lead to the functional saturation of membrane Kv4.3 in interneurons, unlike DGC. Taken together, our findings suggest that TRPC6 may play an important role in the maintenance of neuronal excitability via ERK1/2-mediated membrane Kv4.3 localization in the DG.

In conclusion, the present study provided the first evidence that TRPC6-mediated ERK1/2 activation may subcellular Kv4.3 localization in neurons, which is cause-effect relationship between neuronal excitability and seizure susceptibility. Therefore, TRPC6 will be an interesting and important therapeutic target for epilepsy.

## Author Contributions

T-CK designed and supervised the project. J-EK, J-YP and T-CK performed the experiments described in the manuscript and analyzed the data. J-EK and T-CK wrote the manuscript.

## Conflict of Interest Statement

The authors declare that the research was conducted in the absence of any commercial or financial relationships that could be construed as a potential conflict of interest.
