# Supplementary material for: TRPC6-mediated ERK1/2 Activation Regulates Neuronal Excitability via Subcellular Kv4.3 Localization in the Rat Hippocampus
Source: Front Cell Neurosci. 2017 Dec 20;11:413. doi: 10.3389/fncel.2017.00413 (PMC5742353; doi:10.3389/fncel.2017.00413)
Supplement: Supplementary file 1 [file Data_Sheet_1.pdf]

Supplementary information

TRPC6-mediated ERK1/2 activation regulates neuronal excitability via subcellular Kv4.3 localization in the rat hippocampus

Ji-Eun Kim, Jin-Young Park, Tae-Cheon Kang*

Department of Anatomy and Neurobiology, Institute of Epilepsy Research, College of Medicine, Hallym University, Chuncheon 200-702, South Korea.

Running title: TRPC6-mediated subcellular Kv4.3 localization

* Correspondence should be addressed to T-CK (e-mail: tckang@hallym.ac.kr)

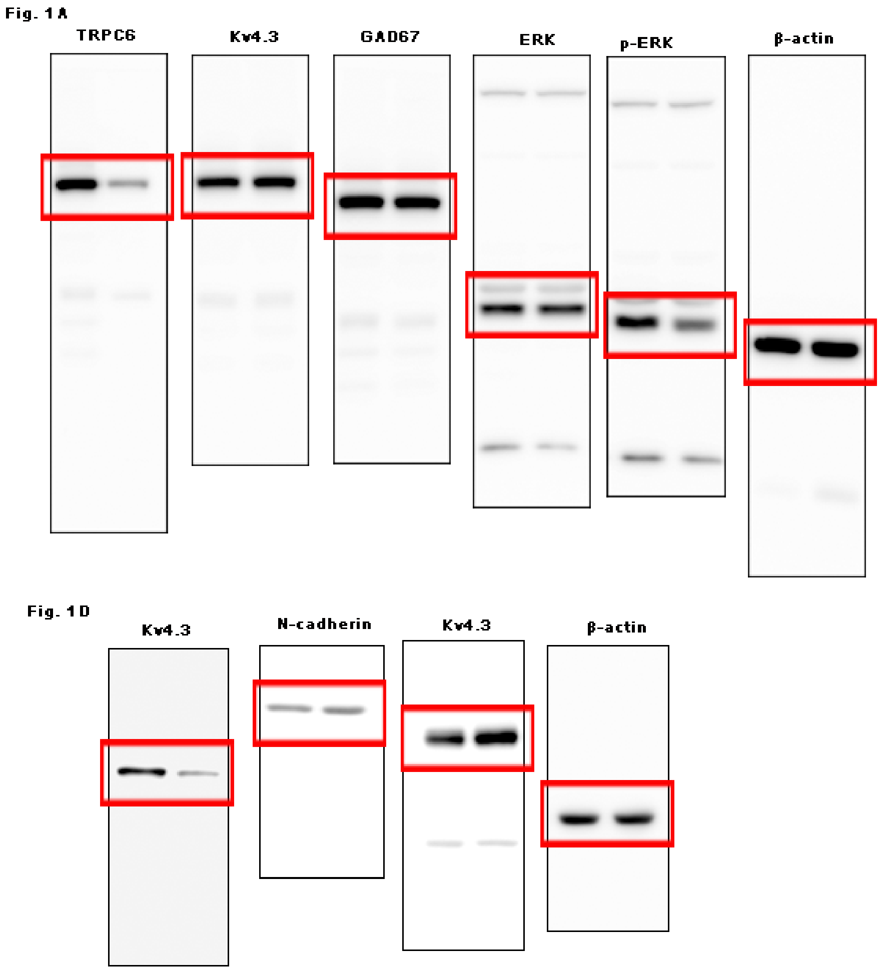

Supplementary Fig. 1. Full-length gel images of western blot data in Fig. 1. The cropped parts of western blots are indicated with boxes.

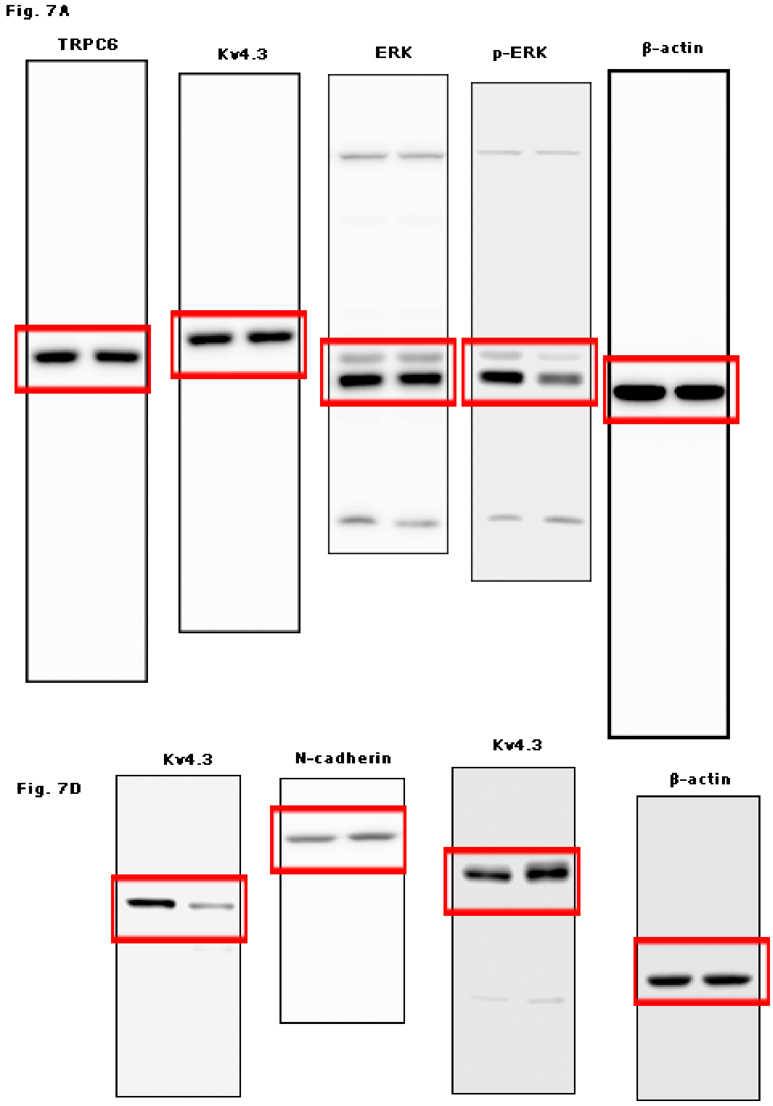

Supplementary Fig. 2. Full-length gel images of western blot data in Fig. 7. The cropped parts of western blots are indicated with boxes.

Fig. 8A

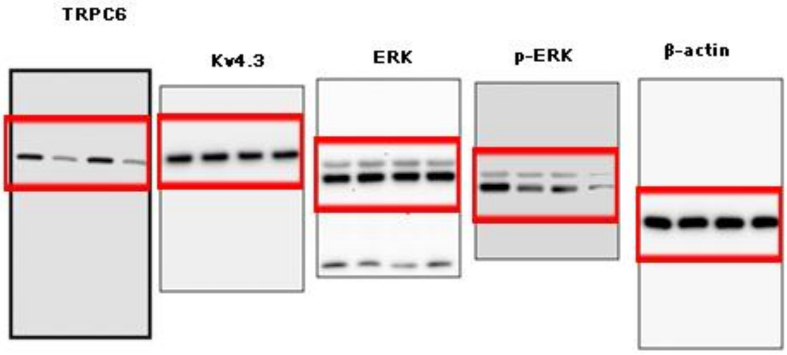

Fig. 8C

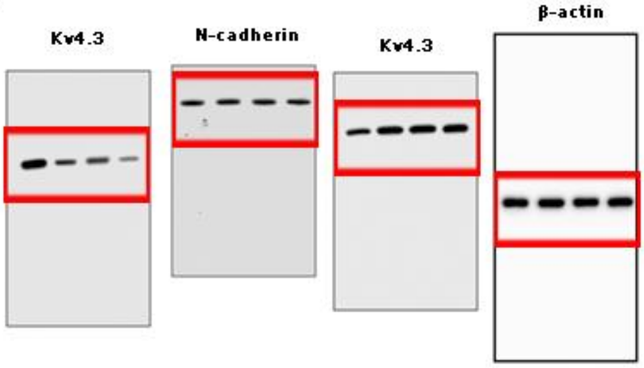

Supplementary Fig. 3. Full-length gel images of western blot data in Fig. 8. The cropped parts of western blots are indicated with boxes.

Fig. 9A

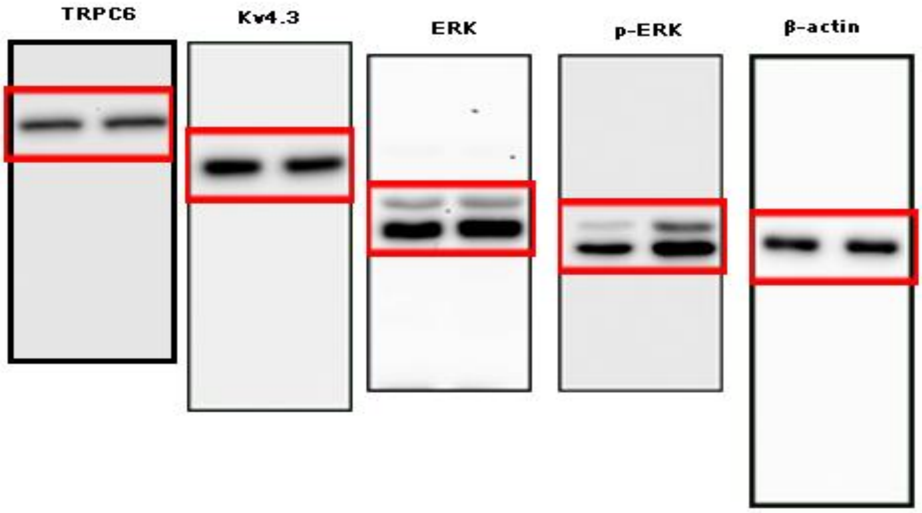

Fig. 9D

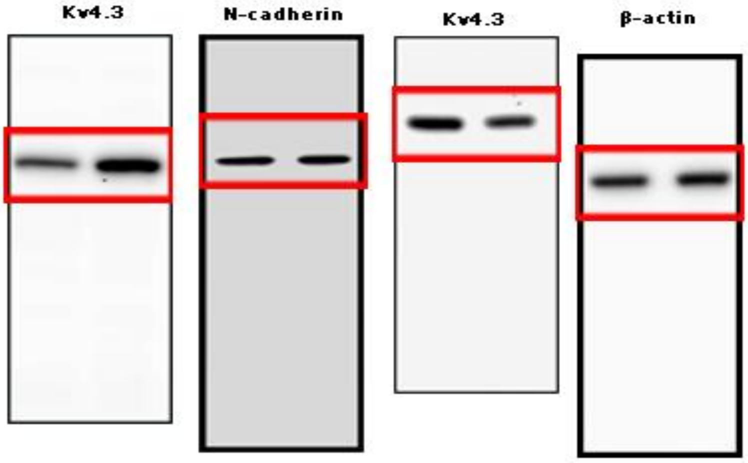

Supplementary Fig. 4. Full-length gel images of western blot data in Fig. 9. The cropped parts of western blots are indicated with boxes.
